# Optimizing Oral Cancer Screening: Latent Class Analysis of Chairside Adjuncts in a High-Risk Dental Cohort

**DOI:** 10.7759/cureus.98157

**Published:** 2025-11-30

**Authors:** Lalitkumar P Gade, Sahrish Tariq, Shreya Bhukal, Radhika Thakkar, Grishmi Niswade, Shreya Chatterjee, Rahul VC Tiwari, Seema Gupta, Manish Sharma

**Affiliations:** 1 Department of Oral Pathology, Sau Mathurabai Bhausaheb Thorat Dental College and Hospital, Sangamner, IND; 2 Department of Public Health Dentistry, Swami Devi Dyal Dental College and Hospital, Panchkula, IND; 3 Department of Cardiology, All India Institute of Medical Sciences, New Delhi, IND; 4 Eastman Institute for Oral Health, University of Rochester School of Medicine and Dentistry, Rochester, USA; 5 Department of Periodontology, Swargiya Dadasaheb Kalmegh Smruti Dental College and Hospital, Nagpur, IND; 6 Department of Laboratory Medicine, Rajendra Institute of Medical Sciences, Ranchi, IND; 7 Department of Dental Research Cell, Dr. D. Y. Patil Dental College and Hospital, Dr. D. Y. Patil Vidyapeeth (Deemed to be University), Pune, IND; 8 Department of Orthodontics, Kothiwal Dental College and Research Centre, Moradabad, IND; 9 Department of Oral Pathology, Jawahar Medical Foundation's Annasaheb Chudaman Patil Dental College, Dhule, IND

**Keywords:** chairside, latent class analysis, oral cancer, screening, tools

## Abstract

Introduction: Oral cancer remains a major public health challenge in high-burden regions, and late diagnosis contributes to poor survival. Conventional oral examination (COE) may be limited by subjectivity and low specificity, prompting the evaluation of adjuncts, such as toluidine blue (TB) staining, autofluorescence imaging, and oral brush cytology. This study applied latent class analysis (LCA) to estimate the unbiased diagnostic performance of multiple chairside tests in a single cohort.

Materials and methods: A cross-sectional diagnostic study was conducted on 100 adults (aged 18-70 years) with visible oral lesions. Sequential testing included COE, VELscope autofluorescence (LED Dental Inc., White Rock, British Columbia, Canada), OralCDx brush cytology (CDx Diagnostics, Suffern, NY, USA), and 1% TB staining, which were performed under standardized conditions by calibrated examiners (kappa > 0.80). Incisional biopsy was performed for lesions that were positive in ≥2 tests. LCA modeled the true disease status without universal verification; traditional metrics used histopathology as a reference.

Results: Histopathology confirmed dysplasia or malignancy in 43 patients (43%). COE showed high sensitivity (83.7%), but low specificity (54.4%). TB staining yielded a sensitivity of 69.8% and specificity of 86.0%, and autofluorescence showed a sensitivity of 60.5% and specificity of 89.5%. Brush cytology achieved a balanced accuracy (sensitivity 83.7%; specificity 80.7%), with the strongest correlation with histopathology (r = 0.64). LCA identified two latent classes, brush cytology and TB, demonstrating superior class discrimination. The inter-test agreement was highest between COE and TB (r = 0.61).

Conclusions: No single test was found to be optimal. Brush cytology offered the best standalone accuracy, whereas COE and autofluorescence served as sensitive initial screens. A two-tier sequential strategy comprising COE/autofluorescence followed by brush cytology maximizes case detection and reduces false positives. The LCA provides robust and unbiased estimates for real-world screening. Integration into routine dental practice with structured training can improve early detection of oral dysplasia in high-risk populations.

## Introduction

Oral cancer, encompassing malignancies of the lips, tongue, and oropharynx, remains a global health burden with over 19.3 million new cases and almost 10 million deaths annually, predominantly in low- and middle-income countries [1]. Despite advances in therapy, five-year survival hovers is 50%-60%, largely due to late-stage diagnosis in 60%-70% of cases [2]. Conventional oral examination (COE) by visual inspection and palpation is the cornerstone of screening in general dental practice, yet its diagnostic odds ratio has been found to be low and poor (6.1), limited by operator experience and lesion subtlety [3]. Chairside non-invasive adjuncts such as toluidine blue (TB) vital staining, chemiluminescence (ViziLite (Zila Pharmaceuticals, Phoenix, AZ, USA), Microlux (AdDent Inc., Danbury, CT, USA)), autofluorescence (VELscope (LED Dental Inc., White Rock, British Columbia, Canada)), and brush cytology (OralCDx (CDx Diagnostics, Suffern, NY, USA)) have been introduced to enhance early detection [4].

A systematic review by Pierfelice et al. [4] evaluated the sensitivity and specificity of various chairside non-invasive oral cancer screening methods in 61 articles and concluded that all methods had low specificity and that incisional biopsy was the best method. Randomized trials and cohort studies have yielded conflicting diagnostic yields, compounded by the absence of a perfect reference standard, as histopathology is invasive and often deferred in benign-appearing lesions, introducing verification bias.

Traditional accuracy metrics (sensitivity and specificity) assume biopsy to be the gold standard; however, in real-world screening, only suspicious lesions are biopsied, skewing estimates. Latent class analysis (LCA) offers a robust alternative by modeling an unobserved “true disease” state using patterns across multiple imperfect tests, yielding unbiased estimates without universal verification [5]. Collins and Huynh [6] estimated the diagnostic test accuracy in the absence of a gold standard using latent variable models. No prior study has applied LCA to assess the efficacy of chairside non-invasive oral cancer screening tests in a single cohort using multiple adjuncts simultaneously.

This cross-sectional diagnostic study aimed to evaluate the efficacy of chairside non-invasive oral cancer screening tests using LCA in a general dentistry patient population. Specific objectives include estimating sensitivity, specificity, and diagnostic odds ratios for COE, TB, autofluorescence, and brush cytology; assessing inter-test agreement; and comparing LCA-derived accuracy against biopsy-confirmed cases. The null hypothesis states that there would be no significant difference in diagnostic accuracy between any adjunct and COE alone and that LCA-based estimates do not differ from those derived using histopathology as the reference standard.

## Materials and methods

Study design and setting

This study employed a cross-sectional diagnostic accuracy design to prospectively evaluate the efficacy of multiple chairside non-invasive oral cancer screening tests in a general dentistry patient population. The study duration spanned from September 2022 to December 2023. Ethical approval was obtained from the institutional ethical committee of the dental college (EC/NEW/INST/2022/2959/SS109) in accordance with the Declaration of Helsinki. Written informed consent was obtained from all patients after providing an information sheet detailing the study aims, procedures, risks (minimal, e.g., transient staining from dyes), benefits (early detection), and voluntary withdrawal rights. Data confidentiality was maintained by using anonymized identifiers with secure storage on password-protected servers.

Sample size estimation

The sample size was calculated using G*Power software (version 3.6.9) (Heinrich-Heine-Universität Düsseldorf, Düsseldorf, Germany) based on the a priori analysis of a one-sample test. An effect size of 0.07 was derived from the study by Kaur and Jacobs [7], which utilized the autofluorescence method for oral epithelial dysplasia screening and reported a sensitivity of 67%. With a statistical power of 80% and a confidence level of 95%, the minimum required sample size for the present study was determined to be 89. To enhance the robustness of the analysis, the sample size was increased to 100 patients.

Eligibility criteria

Inclusion criteria were adults aged 18-70 years who presented for routine dental check-ups or with self-reported oral symptoms (such as persistent lesions > 2 weeks) and at least one clinically visible oral lesion (such as white/red patch, ulcer, or induration) suspicious for potential malignancy or dysplasia based on initial visual inspection. Patients had risk factors for oral cancer (such as tobacco use, alcohol consumption, and betel quid chewing) or were asymptomatic high-risk individuals (such as those aged >40 years with cumulative exposure). The ability to provide informed consent and tolerate non-invasive procedures is required.

Exclusion criteria include pregnant or lactating women, individuals with known allergies to dyes (such as thiazine-based) or anesthetics, acute oral infections or inflammatory conditions (such as active herpes or severe gingivitis) that could confound lesion visualization, previous history of head-neck malignancy or ongoing chemotherapy/radiotherapy, inability to complete follow-up (such as non-residents), or cognitive impairment precluding consent. Lesions < 5 mm or clearly traumatic (such as aphthous ulcers) were excluded after initial screening to focus on potentially malignant epithelial lesions (PMELs).

Methodology

Patients attended a single 45-60-minute screening visit following a four-hour fast to minimize salivary interference. All procedures were performed in a standardized clinical bay under ambient lighting (500-700 lx) by calibrated examiners blinded to the prior test results, where feasible. The protocol sequence minimized carryover bias: (1) COE, (2) autofluorescence, (3) brush cytology, and (4) TB staining, with five-minute intervals and saline rinses between the tests.

COE was performed by a general dentist using white light, a tongue depressor, and gauze for systematic inspection/palpation of 12 intraoral sites (lips, buccal mucosa, floor of mouth, etc.). Lesions were scored as positive (suspicious: irregular borders, induration, and erythema/leukoplakia) or negative (benign), using a dichotomous outcome. The duration of the examination was 5-10 minutes.

Autofluorescence examination was performed using a VELscope VX system. The handpiece emitted blue light (400-430 nm) to excite the tissue fluorophores. The patients were asked to rinse with 10 mL of water for one minute. The examiner viewed the oral cavity in a darkened room (extinguisher light off) for two minutes per site. The loss of autofluorescence (dark/pale areas) indicated positivity. The duration of the examination was five minutes.

Brush cytology was performed using the OralCDx BrushTest kit for transepithelial sampling. The small round brush was rotated firmly on the lesion for 30 rotations (15 clockwise and 15 counterclockwise) to collect full-thickness cells, fixed in preservative solution, and mailed to the manufacturer's laboratory for computer-assisted analysis and pathologist review. The results (negative, atypical, and positive for dysplasia/malignancy) were categorized as positive if atypical/positive.

TB staining was performed using 1% TB solution (OraTest brand, Zila Pharmaceuticals Inc., Phoenix, Arizona, USA), which was applied via a cotton swab to the lesion for 20 seconds, followed by a 1% acetic acid rinse. Dark blue uptake indicates positive staining. Non-suspicious mucosa was used as the control. The duration of the examination was three minutes.

For lesions positive in ≥2 tests or COE, incisional biopsy (under local anesthesia, 2% lignocaine) was performed on-site by an oral surgeon, with samples sent for histopathology (gold standard: dysplasia/malignancy vs. benign/reactive). Follow-up at one month ensured healing and compliance. All test results (dichotomous: positive/negative) were recorded on a standardized case report form, including demographics, risk factors, and lesion characteristics (size, site, and duration).

Calibration

To ensure inter- and intraexaminer reliability, five general dentists and one oral pathologist underwent a two-day training workshop prior to patient recruitment. The calibration process included didactic sessions on standardized test protocols and the recognition of PMELs using 50 archived clinical images and videos, followed by hands-on simulation of 20 volunteer models with benign oral lesions. All examiners performed standardized scoring under the supervision of a reference expert, achieving an inter-rater agreement of kappa > 0.80 for COE and all adjunctive tests. Intrarater reliability was maintained throughout the study through monthly blinded repeat scoring of 10% of duplicate cases, with kappa values consistently ≥0.75 across examiners. Blinding was strictly enforced; each examiner was unaware of prior test results during sequential assessments, and histopathology reviewers remained blinded to all screening outcomes.

Statistical analysis

Data analysis was conducted using the Statistical Package for the Social Sciences (SPSS) software (version 25, IBM Corp., Armonk, NY, USA). Categorical variables are summarized as frequencies and percentages. Independent associations between variables were assessed using the chi-squared test. The correlation between the diagnostic tests was evaluated using Spearman’s rank correlation. The diagnostic metrics for each test were reported in terms of sensitivity, specificity, positive predictive value (PPV), and negative predictive value (NPV). In the absence of a definitive gold standard, LCA was used to efficiently estimate the diagnostic accuracy of each test. Statistical significance was set at p ≤ 0.05.

To address partial verification bias and estimate diagnostic accuracy without assuming biopsy as a perfect gold standard, LCA was performed using R (poLCA package, version 1.x). All four screening tests (COE, TB staining, autofluorescence, and brush cytology) were included as dichotomous indicators. A series of 1-4 class models were fitted, and model selection was based on Bayesian Information Criterion (BIC), Akaike Information Criterion (AIC), likelihood ratio (G²), and entropy. The two-class solution demonstrated the best parsimony and interpretability. Local independence was assumed, and model convergence was achieved using 500 iterations and 50 random starts to avoid local minima. Conditional response probabilities (CRPs) for each screening test within each latent class were extracted to estimate class-specific sensitivity and specificity.

## Results

Table 1 presents the descriptive analysis of the study variables, comparing the cases with and without dysplasia. Among the patients, men predominated in both groups, with no significant sex difference (p = 0.58). Thirty-seven (86.05%) patients with dysplasia reported chewing tobacco compared with 41 (71.93%) patients without dysplasia, although the association was not statistically significant (p = 0.09). Similarly, smoking was more prevalent among patients with dysplasia than among those without dysplasia, approaching significance (p = 0.07). The most common lesion site was the buccal mucosa in both groups. The lesion size distribution did not differ significantly between groups (p = 0.53). However, lesion color showed a highly significant association (p = 0.001), with mixed-colored lesions predominating in dysplasia cases, whereas white lesions were more frequent in non-dysplastic cases. The surface texture showed a statistically significant trend toward rougher surfaces in dysplastic lesions (p = 0.04).

**Table 1:** Distribution of demographic, behavioral, and clinical lesion characteristics among patients with and without histopathologically confirmed dysplasia. *p < 0.05 denotes statistical significance using the chi-squared test. Data are presented as frequency (n) and percentage (%), where n denotes the number of patients.

Parameters	Categories	No dysplasia (n = 57, 57%)	Dysplasia (n = 43, 43%)	Chi-stats	p-value
		Frequency (%)	Frequency (%)		
Sex	Female	23 (40.35)	15 (34.88)	0.34	0.580
	Male	34 (59.65)	28 (65.12)		
Chewing tobacco	No	16 (28.07)	6 (13.95)	2.85	0.090
	Yes	41 (71.93)	37 (86.05)		
Smoking	No	45 (78.95)	27 (62.79)	3.17	0.070
	Yes	12 (21.05)	16 (37.21)		
Site of lesion	Buccal mucosa	33 (57.90)	25 (58.14)	5.22	0.270
	Floor of mouth	5 (8.77)	7 (16.28)		
	Gingiva	11 (19.30)	3 (6.98)		
	Palate	4 (7.02)	6 (13.95)		
	Tongue	4 (7.02)	2 (4.65)		
Size of lesion	Large	4 (7.02)	4 (9.30)	1.28	0.530
	Medium	24 (42.11)	22 (51.16)		
	Small	29 (50.88)	17 (39.54)		
Color of lesion	Mixed	6 (10.53)	18 (41.86)	19.82	0.001*
	Red	4 (7.02)	8 (18.61)		
	White	47 (82.46)	17 (39.54)		
Surface of lesion	Rough	10 (17.54)	14 (32.56)	3.03	0.040*
	Smooth	47 (82.46)	29 (67.44)		

Table 2 shows the association between various screening tests and the histopathological gold standard for detecting dysplasia. COE revealed dysplasia in 36 (83.72%) cases as clinically positive, while only seven (16.28%) cases were clinically negative, showing a statistically significant association (p = 0.001). TB staining demonstrated a strong correlation, with 30 (69.77%) positive cases confirmed as dysplastic compared with 13 (30.23%) negative cases (p = 0.001). Similarly, autofluorescence examination showed the highest diagnostic correlation with 36 (83.72%) fluorescence-positive lesions versus seven (16.28%) fluorescence-negative lesions (p = 0.001). The brush cytology method showed dysplasia in 26 (60.47%) positive cases that were histologically confirmed as dysplastic (p = 0.001). All four screening methods showed statistically significant associations with histopathological findings, indicating their reliability in identifying dysplastic lesions, with autofluorescence examination demonstrating the strongest correlation.

**Table 2:** Cross-tabulation of conventional oral examination (COE), toluidine blue staining, brush cytology, and autofluorescence examination results against histopathological diagnosis of dysplasia. *p < 0.05 denotes statistical significance using the chi-squared test. Data are presented as frequency (n) and percentage (%), where n denotes the number of patients.

Parameters	Categories	Histologically		Chi-stats	p-value
		Dysplasia absent	Dysplasia present		
Conventional oral examination	No	31 (54.39)	7 (16.28)	15.11	0.001*
	Yes	26 (45.61)	36 (83.72)		
Toluidine blue staining	No	49 (85.97)	13 (30.23)	32.31	0.001*
	Yes	8 (14.04)	30 (69.77)		
Brush cytology	No	51 (89.47)	17 (39.54)	28.09	0.001*
	Yes	6 (10.53)	26 (60.47)		
Autofluorescence examination	No	46 (80.70)	7 (16.28)	40.84	0.001*
	Yes	11 (19.30)	36 (83.72)		

Table 3 presents the diagnostic performance of various screening tests compared with the histopathological gold standard. COE showed a high sensitivity (83.7%) but relatively low specificity (54.4%), indicating more false positives. TB staining demonstrated balanced sensitivity (69.8%) and high specificity (86%), whereas autofluorescence examination showed the highest specificity (89.5%) with moderate sensitivity (60.5%). The brush cytology method exhibited both high sensitivity (83.7%) and specificity (80.7%), along with the highest NPV (86.8%). Among the screening methods, the brush cytology method showed the best overall diagnostic accuracy, followed by TB staining, indicating its reliability in identifying dysplastic lesions.

**Table 3:** Sensitivity, specificity, positive predictive value (PPV), and negative predictive value (NPV) of each screening test using histopathology as the reference standard. Values expressed in percentages (%).

Diagnostic test	Sensitivity (%)	Specificity (%)	PPV (%)	NPV (%)
Conventional oral examination	83.7	54.4	58.1	81.6
Toluidine blue staining	69.8	86.0	78.9	79.0
Autofluorescence examination	83.7	80.7	76.6	86.8
Brush cytology	60.5	89.5	81.3	75.0

COE, TB staining, and autofluorescence examination have relatively low specificities (54%-86%), indicating that they have a high false-positive rate. Using these alone would lead to many unnecessary biopsies. The brush cytology method has a lower sensitivity (60.5%) than some other methods, meaning that it could miss some true dysplasias if used alone. Therefore, no single test is reliable enough on its own. Although its sensitivity is not the highest, the brush cytology method has a far superior specificity (89.5%). This means that a positive brush cytology result is a strong indicator of "true dysplasia." The optimal strategy, supported by the model, would be to first use a highly sensitive test (such as COE or autofluorescence examination) to cast a wide net and not miss potential cases. Any lesion that tests positive with this first test should then be evaluated using a highly specific brush cytology method. This sequential combined approach maximizes the detection of true dysplasia while minimizing false positives, making the screening process both effective and efficient.

**Figure 1:**
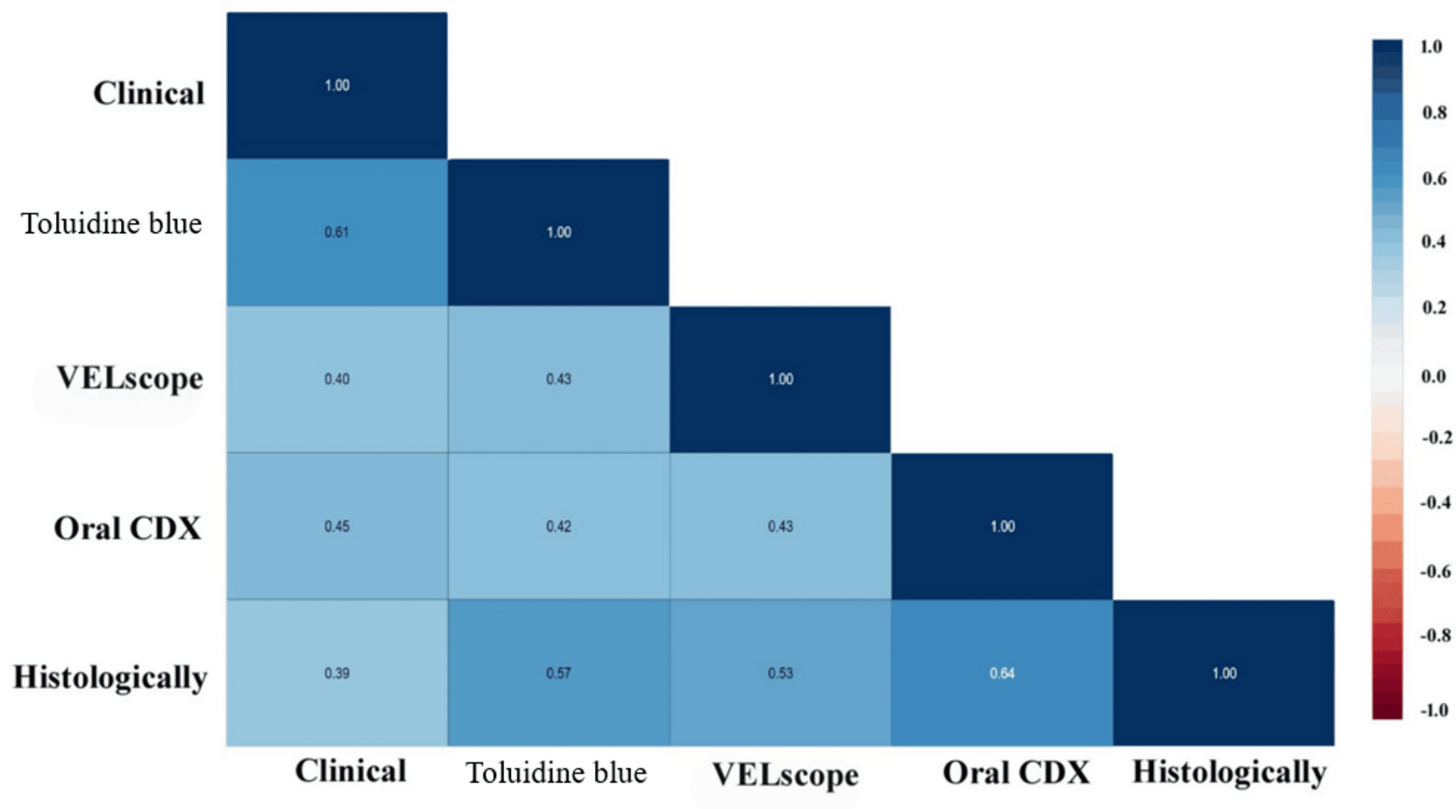
Spearman’s rank correlation coefficients between conventional oral examination (COE), toluidine blue staining, brush cytology (Oral CDx), autofluorescence examination (VELscope), and histopathological diagnosis. Correlation strength: |r| > 0.6 = strong; 0.4–0.6 = moderate; <0.4 = weak.

Figure 1 depicts the correlation analysis between the various screening tests and the histopathological gold standard. The strongest positive correlation with histopathological diagnosis was observed for brush cytology (r = 0.64), followed by TB (r = 0.57) and autofluorescence examination (r = 0.53). COE showed a moderate correlation (r = 0.39) with the histopathological findings. Among the screening tests, the highest inter-test correlation was found between the COE and TB staining tests (r = 0.61). The brush cytology method demonstrated the highest correlation with the gold standard, indicating superior diagnostic accuracy. TB staining and autofluorescence examination also showed moderate-to-strong associations, supporting their utility as reliable adjunctive screening tools.

**Figure 2:**
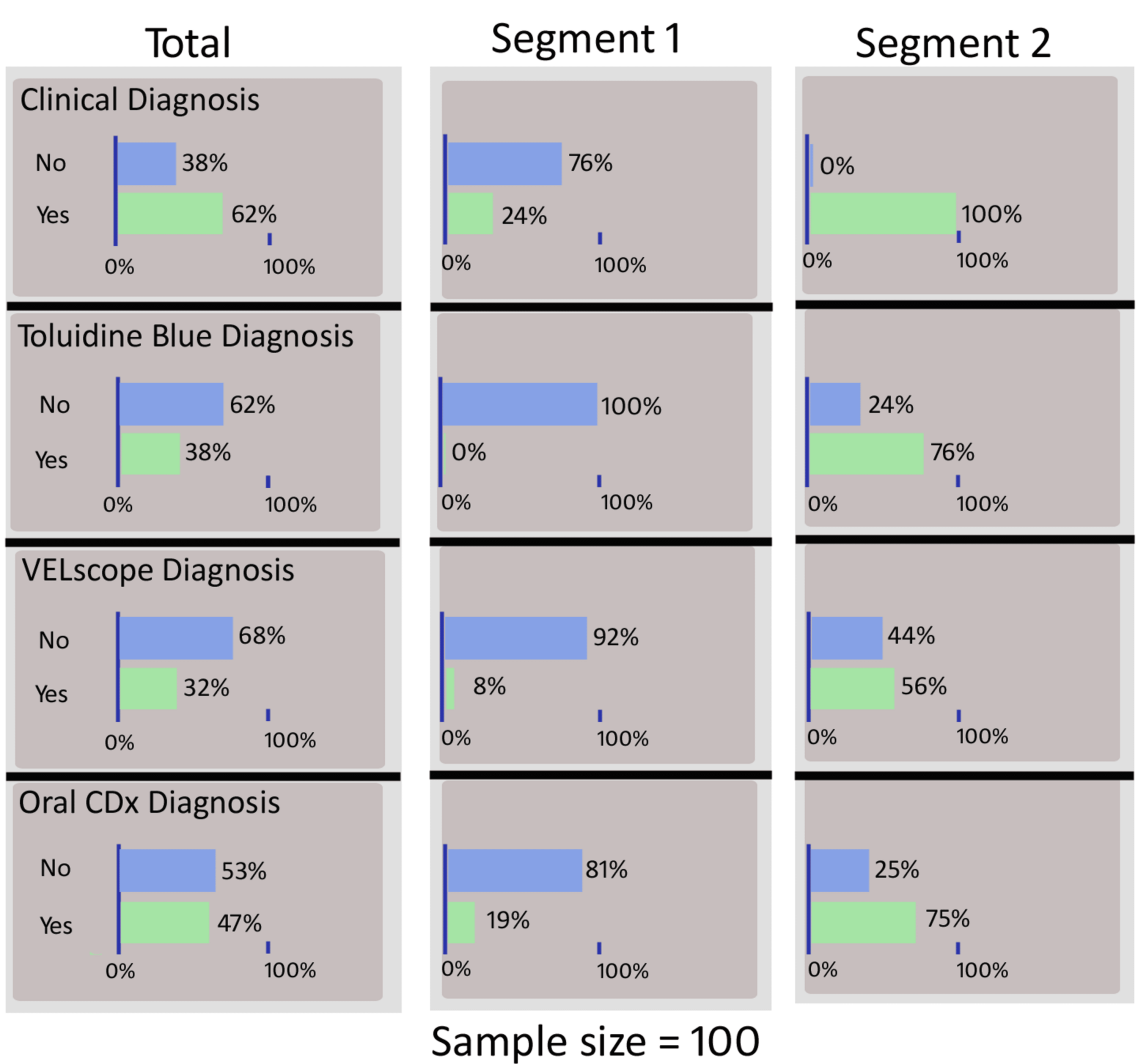
Latent class model identifying two unobserved disease states (non-dysplastic and dysplastic) based on conditional probabilities of test positivity across conventional oral examination (COE), toluidine blue, autofluorescence (VELscope), and brush cytology. Segment 1: non-dysplastic latent class; segment 2: dysplastic latent class. Values represent % test-positive within each latent class.

Figure 2 presents the LCA performed to assess diagnostic test performance in the absence of a feasible gold standard. Two latent segments were identified: segment 1 (non-dysplastic) and segment 2 (dysplastic). In segment 1, most cases were negative across all tests, with 100% negative by TB and 92% by autofluorescence examination. In contrast, segment 2 showed a higher proportion of positives: 100% by clinical diagnosis, 76% by TB, 56% by autofluorescence examination, and 75% by the brush cytology method. LCA indicated that all screening methods effectively differentiated latent classes, with brush cytology and TB showing the strongest discrimination ability.

## Discussion

The present cross-sectional study evaluated the diagnostic efficacy of COE and three chairside non-invasive adjuncts, TB staining, autofluorescence (VELscope), and brush cytology (OralCDx), in a general dental population using both histopathology and LCA. Of the 100 patients harboring clinically visible oral lesions, 43 were histopathologically confirmed as dysplastic or malignant, yielding a prevalence of 43%. This aligns with the high-burden settings in South India, where tobacco-related PMELs are endemic [8]. The male predominance and buccal mucosa as the primary site reflect regional betel quid and smokeless tobacco habits, which are consistent with previous studies [8,9]. The higher prevalence rate observed in our study could be due to the inclusion criteria, in which patients were selected if they had white patch lesions.

When benchmarked against histopathology, COE demonstrated high sensitivity (83.7%) but modest specificity (54.4%), corroborating its role as a broad screening tool that is prone to false positives due to operator subjectivity and lesion subtlety [3]. TB staining achieved balanced metrics (sensitivity 69.8%; specificity 86.0%), replicating meta-analytic pooled estimates (sensitivity 84%; specificity 70%) from 14 studies [10]. Autofluorescence exhibited the highest specificity (89.5%), but lower sensitivity (60.5%), mirroring a systematic review reporting specificity > 90% and sensitivity < 50% in low-prevalence cohorts [11]. Rashid and Warnakulasuriya [11] reported that the autofluorescence method may detect erythematous benign inflammatory lesions, resulting in false-positive results. Nagi et al. [12], in a systematic review, reported 22%-100% sensitivity and 16%-100% specificity with the autofluorescence method.

Brush cytology was superior overall (sensitivity 83.7%, specificity 80.7%, and NPV 86.8%), outperforming earlier reports of 87%-100% sensitivity, 95%-100% specificity, and 98% NPV [13], likely attributable to transepithelial sampling and computer-assisted analysis, reducing sampling error. Walsh et al. [10] reported 91% specificity and sensitivity in 12 studies using oral cytology. The utilization of liquid-based cytology mitigates the issues associated with sampling and fixation, thereby offering enhanced cytological morphology. However, certain disadvantages of this method are that lesions that are diminutive or less conspicuous may be disregarded, challenges in identifying lesions in the presence of necrotic tissue or coagulated hemoglobin, and insufficient education of practitioners [10].

Inter-test correlations underscored complementary strengths: COE-TB staining and brush cytology-histopathology were strongest, suggesting that TB staining enhances COE specificity, while cytology provides cytomorphometric confirmation. The sequential strategy of COE/autofluorescence followed by brush cytology mitigates individual limitations, akin to combined VELscope-TB staining protocols that reduce unnecessary biopsies by 55% in high-risk cohorts [14]. Kaur and Jacobs [7] reported that a combined approach of salivary protoporphyrin X and the VELscope system led to increased sensitivity and specificity for diagnosing PMELs. The principle underlying tissue autofluorescence pertains to alterations in the structural composition and metabolic processes of the epithelium, in conjunction with modifications in the subepithelial stroma, which subsequently affect their interaction with electromagnetic radiation [15]. TB, a cationic metachromatic dye, selectively stains dysplastic/malignant oral lesions owing to increased nuclear DNA/RNA, widened intercellular spaces, and higher acidic components in the abnormal epithelium. After application and acetic acid rinsing, dark blue uptake indicated positivity, whereas normal mucosa remained unstained [16].

LCA circumvented the verification bias inherent in partial biopsy referral, modeling an unobserved “true disease” state. Two latent classes emerged: non-dysplastic (100% TB staining-negative, 92% autofluorescence-negative) and dysplastic (100% COE-positive, 75% brush cytology-positive). LCA-derived sensitivities exceeded biopsy-based estimates for the TB staining method (76% vs. 69.8%) and brush cytology (75% vs. 83.7%), while specificities converged, validating LCA’s utility when universal verification is unethical [5,6]. This is the first application of LCA as a simultaneous adjunct in oral cancer screening. Risk factor analysis revealed that lesion color (mixed red-white) and rough texture were significant dysplasia predictors, reinforcing clinical indices such as the oral potentially malignant disorder scoring system [17]. Near-significant tobacco associations likely reflect the selected high-risk cohort, where cumulative exposure homogenizes the risk [18].

Clinical implications and recommendations

No single test is sufficient for standalone screening. A two-tier algorithm is recommended: initial COE or autofluorescence to maximize case detection, followed by brush cytology for a high-specificity triage. Positive cytology warrants immediate biopsy, whereas dual-negative results permit six-month surveillance. Integration into general dental practice can reduce late-stage diagnoses by 20%-30%. Training modules that emphasize sequential protocols and LCA-informed decision thresholds are recommended for calibration.

Strengths

The study incorporates several methodological strengths, including the use of standardized and calibrated examiners (κ > 0.80), a systematic multitest evaluation of four widely used chairside adjuncts, and the application of LCA to address partial verification bias when universal biopsy was not feasible. The prospective design, predefined testing sequence, and structured data collection enhance procedural consistency. Although not entirely novel, the concurrent assessment of multiple adjunctive tests within a single cohort provides practical comparative insights that can inform clinical decision-making in high-risk populations.

Limitations

This study has several important limitations. As a single-center study with a modest sample size, generalizability is limited, and prevalence patterns unique to the study setting may influence diagnostic accuracy estimates. Although biopsy was used selectively based on predefined criteria, this partial-verification approach introduces potential verification and spectrum bias. Earlier versions of the manuscript did not clearly articulate all biopsy referral criteria, which we have now clarified. Important methodological constraints include the possibility of inconsistencies between LCA-derived and biopsy-derived accuracy estimates, and the assumption of local independence within the LCA model, which may not fully reflect real-world inter-test correlation. Dichotomizing multilevel test outputs (e.g., atypical vs. positive cytology) may also contribute to misclassification. Despite examiner calibration, some degree of operator-dependent variability is unavoidable. These limitations should be considered when interpreting the results and the proposed sequential testing strategy.

## Conclusions

This study demonstrated that no single chairside test was sufficient for oral cancer screening. Brush cytology exhibited superior diagnostic accuracy (sensitivity 83.7%; specificity 80.7%), followed by TB and autofluorescence, whereas COE offered high sensitivity but low specificity. LCA validated unbiased estimates, confirming the test complementarity. A sequential approach, comprising COE/autofluorescence for initial screening, followed by brush cytology for confirmation, will optimize detection and minimize unnecessary biopsies. Implementing this algorithm in general dental practice with standardized training can enhance the early detection of dysplasia in high-risk populations. Multicenter longitudinal studies are recommended to assess the progression risk and cost-effectiveness.
